# Toxicity and antitumor potential of *Mesosphaerum sidifolium* (Lamiaceae) oil and fenchone, its major component

**DOI:** 10.1186/s12906-017-1779-z

**Published:** 2017-07-03

**Authors:** Thaísa Leite Rolim, Déborah Ribeiro Pessoa Meireles, Tatianne Mota Batista, Tatyanna Kelvia Gomes de Sousa, Vivianne Mendes Mangueira, Renata Albuquerque de Abrantes, João Carlos Lima Rodrigues Pita, Aline Lira Xavier, Vicente Carlos Oliveira Costa, Leônia Maria Batista, Josean Fechine Tavares, Marcelo Sobral da Silva, Marianna Vieira Sobral

**Affiliations:** 1 Programa de Pós-graduação em Produtos Naturais e Sintéticos Bioativos, Centro de Ciências da Saúde, Universidade Federal da Paraíba, João Pessoa, Paraíba 58051-970 Brazil; 2 Departamento de Ciências Farmacêuticas, Universidade Federal da Paraíba, João Pessoa, Paraíba 58051-970 Brazil

**Keywords:** *Mesosphaerum sidifolium*, Lamiaceae, Fenchone, Hemolysis, Ehrlich ascites carcinoma, Genotoxicity

## Abstract

**Background:**

The essential oil from *Mesosphaerum sidifolium *(L’Hérit.) Harley & J.F.B.Pastore (syn. *Hyptis umbrosa*), Lamiaceae (EOM), and its major component, have been tested for toxicity and antitumor activity.

**Methods:**

EOM was obtained from aerial parts of *M. sidifolium* subjected to hydro distillation, and gas chromatography-mass spectrometry was used to characterize the EOM chemical composition. The toxicity was evaluated using haemolysis assay, and acute toxicity and micronucleus tests. Ehrlich ascites carcinoma model was used to evaluate the *in vivo* antitumor activity and toxicity of EOM (50, 100 and 150 mg/kg), and fenchone (30 and 60 mg/kg) after 9 d of treatment.

**Results:**

The EOM major components were fenchone (24.8%), cubebol (6.9%), limonene (5.4%), spathulenol (4.5%), β-caryophyllene (4.6%) and α-cadinol (4.7%). The HC50 (concentration producing 50% haemolysis) was 494.9 μg/mL for EOM and higher than 3000 μg/mL for fenchone. The LD50 for EOM was approximately 500 mg/kg in mice. The essential oil induced increase of micronucleated erythrocytes only at 300 mg/kg, suggesting moderate genotoxicity. EOM (100 or 150 mg/kg) and fenchone (60 mg/kg) reduced all analyzed parameters (tumor volume and mass, and total viable cancer cells). Survival also increased for the treated animals with EOM and fenchone. For EOM 150 mg/kg and 5-FU treatment, most cells were arrested in the G0/G1 phase, whereas for fenchone, cells arrested in the S phase, which represents a blockage in cell cycle progression. Regarding the toxicological evaluation, EOM induced weight loss, but did not induce hematological, biochemical or histological (liver and kidneys) toxicity. Fenchone induced decrease of AST and ALT, suggesting liver damage.

**Conclusions:**

The data showed EOM caused in vivo cell growth inhibition on Ehrlich ascites carcinoma model by inducing cell cycle arrest, without major changes in the toxicity parameters evaluated. In addition, this activity was associated with the presence of fenchone, its major component.

## Background

Cancer is a group of diseases characterized by the uncontrolled cells growth and multiplication that can invade diverse tissues. In this context, carcinogenesis is a multi-step process that begins with a transformation of normal cells into cancer cells, progresses with hyperproliferation and culminates in the acquisition of angiogenic properties, invasive potential, and establishment of metastatic lesions [1]. Nowadays, it is an important public health problem worldwide, since it is a leading cause of mortality and morbidity in the Western countries and the second leading cause of death in third world countries, thus imparting a significant societal burden [2, 3].

Natural products continue to be important source of cancer-fighting compounds [4]. Among them, essential oils from plants have been useful in cancer prevention and treatment [5–7]. Its effects are associated with antioxidant, antimutagenic, antiproliferative, and immunostimulatory properties, besides modulating multidrug resistance [8].

The family Lamiaceae is composed of herbs, shrubs and trees, with about 300 genera and 7500 species, having a cosmopolitan distribution, but mainly centered in the Mediterranean region, where is an integral part of the vegetation. In Brazil, there are 28 genera and about 350 species [9].

*Mesosphaerum* species have shown antimicrobiane [10, 11], antiulcer [12], antidepressive [13], anti-inflammatory and antinociceptive [14, 15], and antihypertensive activities [14]. Recent data showed that the *Hyptis mutabilis* aqueous extract has antitumor activity against sarcoma 180 (murine tumor), and low toxicity. It was also observed that its hexane extract showed moderate inhibition of Ehrlich solid tumor [16].

*Mesosphaerum sidifolium* (L’Hérit.) Harley & J.F.B.Pastore (syn. *Hyptis umbrosa)* (Lamiaceae) is popularly known as “aleluia do serrote” [17] and “alfazema do mato” [18]. The most commonly used parts are the leaves and flowers. In folk medicine, *M. sidifolium* is used in stomach disorders and headaches treatment, besides of its use as expectorant, carminative and tonic [19]. However, there are few reports in the literature on *M. sidifolium*. This study determined the chemical composition, toxicity and anti-tumour activity of the essential oil from *M. sidifolium* aerial parts (EOM), and its major component.

## Methods

### Drugs and reagents

Propidium iodide (P4170 Sigma-Aldrich), 5-Fluorouracil (5-FU) (F6627 Sigma-Aldrich), Triton X-100 (93,443 Sigma-Aldrich), Tween 80 (P4780 Sigma-Aldrich), and cyclophosphamide (C7397 Sigma-Aldrich), Dimethylsulfoxide (DMSO) (67–68-5 Mallinckrodt Chemicals®), Sodium thiopental (Thiopentax®) was purchased from Cristália (Itapira, SP, Brazil), and heparin (Parinex®) from Hipolabor (Sabará, MG, Brazil). Kits for biochemical and hematological analysis were purchased from LABTEST® (ALT/GPT Liquiform ref.: 108; ALT/GPT Liquiform ref.:1008; Creatinina ref.: 35; Uréia CE ref.: 27) (Lagoa Santa, MG, Brazil). (+)-Fenchone (analytical standard) (46,208 Sigma-Aldrich).

### Plant material

Aerial parts of *Mesosphaerum sidifolium* (L’Hérit.) Harley & J.F.B.Pastore *(syn. Hyptis umbrosa*) were collected in July 2015 in Maturéia, Paraíba state, Brazil. The species was identified by Dr. Maria de Fatima Agra. Voucher specimen number AGRA 6964 was deposited at the Herbarium Lauro Pires Xavier at the Federal University of Paraíba, Brazil.

### Extraction of the essential oil

The fresh leaves of *M. sidifolium* were submitted to hydrodistillation for 4 h using a Clevenger-type apparatus at 40 °C. The oil obtained has a yellow color which was dried using anhydrous sodium sulfate and filtered afterwards. For further analysis, 2 μL of the volatile oil obtained was dissolved in 1 mL of ethyl acetate.

### Analysis of essential oil

The GC analysis was performed on a Shimadzu QP2000-PLUS-A gas chromatograph using fused silica capillary column DB-5 (30 mx 0.25 mm id, 0.25 mM film thickness). Helium was used as carrier gas at a flow rate of 1.0 mL/min. The oven temperature was programmed from 60° to 240° at 3 °C/min. The injector and detector temperatures were 220 °C and 230 °C, respectively.

### Gas chromatography - mass spectrometry (GC-MS)

Analysis by Gas Chromatography - Mass Spectrometry (GC-MS) was performed on a Shimadzu QP2000-PLUS system-Quadrupole MS, operating with ionization energy of 70 eV and fused silica capillary column DB-5 (30 mx 0.25 mm id, 0.25 mM film thickness) with helium as a carrier gas at a flow rate of 1 mL/min with a split. The temperatures of injector and detector were 220 °C and 230 °C, respectively. The column temperature was set from 60 °C to 240 °C at 3 °C/min. The substances identification was performed by comparing their mass spectra with the GC-MS database (62 Nist Research Library) and Kovats retention index [20]. Retention rates of the compounds were obtained by coinjection of the essential oil with a standard mixture of hydrocarbons (C9-C24), applying the equation of Van den Dool & Kratz [21].

### Tumor cell line

Ehrlich carcinoma cell line was generously provided by Pharmacology and Toxicology Division, CPQBA, UNICAMP (Paulínia, SP, Brazil). The cells were maintained in the peritoneal cavities of Swiss mice in the Dr. Thomas George Bioterium (Research Institute in Drugs and Medicines/Federal University of Paraíba, Brazil).

### Animals

Swiss albino mice (*Mus musculus*), females (27–31 g), obtained from the Dr. Thomas George Bioterium (Research Institute in Drugs and Medicines/Federal University of Paraíba, Brazil), were used. The animals were randomly housed in cages containing six animals with food and water ad libitum. All animals were kept on a 12 h/12 h off light-dark cycle (lights on at 6:00 a.m.). All procedures were previously approved by the Animal Studies Committee from the Federal University of Paraíba (CEUA-UFPB, no. 0510/11).

### Pharmacological assays

#### Evaluation of cytotoxicity against mice erythrocytes

EOM cytotoxicity activity and its major component was evaluated using mice erythrocytes [22]. Briefly, fresh blood samples were collected, and re-suspended in PBS to make a 0.5% (*v*/v) solution. Various concentrations of EOM (0–1000 μg/mL for EOM, and 0–3000 μg/mL for its major component) dissolved in DMSO (5% *v*/v in PBS), were added to the red blood cells suspension. The plates with the sample-erythrocyte mixtures were incubated in a mixer for 60 min and then centrifuged. The supernatant was carefully removed. After removal, 200 μL of a solution of Triton X-100 (0.1%) was added to each well containing the sample-erythrocyte mixtures and thoroughly stirred. The hemolysis caused was determined by spectrophotometry at 415 nm. The concentration that produced 50% hemolysis (HC50) was then determined. Positive control (100% hemolysis), and negative control (0% hemolysis) incubated erythrocytes with 0.1% Triton X-100 in PBS, and 5% DMSO in PBS, respectively, were used.

### Evaluation of acute preclinical toxicity

The evaluation of acute preclinical toxicity for EOM was performed based on the “Guidelines for Testing of Chemicals” n° 423 from The Organisation for Economic Co-operation and Development [23]. Mice (*n*=3 females/group) were subjected to single doses of 300 or 2000 mg/kg of EOM intraperitoneally (i.p.), to the control group it was administered vehicle alone (5% (*v*/v) Tween 80 in saline). For toxicity detection, suggestive signs of Central Nervous System (CNS), or Autonomic Nervous System (ANS) activity were recorded. Careful observation was performed at the intervals: 0, 15, 30, and 60 min, after 4 h, and daily for 14 days. The dose responsible for the death of 50% of the experimental animals (LD_50_) was estimated.

### Evaluation of genotoxicity

For the micronucleus assay, female mice (*n*=5/group) were treated (i.p.) with single dose of 150 or 300 mg/kg EOM [24]. A positive control group (cyclophosphamide at 50 mg/kg i.p.), and a negative control group (Tween 80 at 5% in saline), were included. After 48 h, the animals were anesthetized with sodium thiopental (40 mg/kg), and peripheral blood samples were obtained from the tail (10 μL), for the blood smears. For each animal, three blood smears were prepared, and a minimum of 2000 erythrocytes were counted to determine the number of micronucleated erythrocytes [25].

### Evaluation of in vivo antitumor activity

Five to seven-day-old Ehrlich tumor cells, 0.5 mL at 2.0 × 10^6^ cells/mL, were implanted in the peritoneal cavity of the female mice (*n*=6/group) [26]. One day after inoculation, EOM (50, 100 or 150 mg/kg) and its major component (30 or 60 mg/kg) were dissolved in 5% (*v*/v) Tween-80 in 0.9% (*w*/*v*) NaCl, and administered for nine consecutive days (i.p.). 5-FU (25 mg/kg) was used as a positive control. The healthy group (healthy mice) and tumor control group (mice bearing Ehrlich ascites carcinoma cells), were treated with 5% Tween-80 in 0.9% (*w*/*v*) NaCl. On the eleventh day, mice were kept fasting for 6 h, and peripheral blood samples from all groups were collected from the retro-orbital plexus under light sodium thiopental anesthesia (40 mg/kg), the animals were then euthanized.

The volume of ascitic fluid collected from the peritoneal cavity was expressed in milliliter (mL). It was determined the total viable cancer cells by trypan blue assay [26]. Tumor weights were measured by taking the mice’s weights before and after the collection of the ascitic fluid from peritoneal cavity, and expressed in grams (g).

The remaining animals (*n*=6/group) were kept alive with food and water ad libitum to calculate the animal’s survival rates.

### Cell cycle analyses

For the determination of cell cycle phase distribution, 10^6^ cells from ascitic fluid of treated animals (groups: tumor control; 50, 100 and 150 mg/kg EOM; 30 and 60 mg/kg major component) were centrifuged at 230 *g* for 7 min. The supernatant was removed and the pellet was resuspended in 0.3 mL of hypotonic fluorocromic solution containing RNase (0,5 mg/mL), Triton-X (0,25%) and propidium iodide (PI) (0,25 mg/mL). Then, the analysis was performed by cytometric flow (BD FACSCalibur®, USA), a total of 10,000 events were obtained, and data were analyzed using WinMDI 2.9 software [27].

### Toxicity evaluation for transplanted mice

Body weights were registered at the beginning and end of the treatment while the water and food consumption was evaluated daily for the nine days of the treatment. Liver, spleen, thymus, and kidneys were weighed for the determination of their organ indices [organ weight (mg)/animal weight (g)]. For biochemical analysis, serum samples were used to determine the levels of urea, creatinine, and the activities of alanine aminotransferase (ALT) and aspartate aminotransferase (AST). For the hematological analysis, heparinized whole blood was used to determine: hemoglobin (Hb) level, red blood cell (RBC) count, hematocrit (Hct), and the red cell indices mean corpuscular volume (MCV), mean corpuscular hemoglobin (MCH), and mean corpuscular hemoglobin concentration (MCHC) and, total and differential leukocyte counts. After weight determination and fixation in 10% (*v*/v) formaldehyde, portions of the livers and kidneys were cut into small pieces, then into sections of 3 μm, and stained with hematoxylin-eosin, and examined microscopically for lesions [28].

### Statistical analysis

Data are presented as mean ± SEM. The differences between experimental groups were compared by analysis of variance (ANOVA), followed by Tukey’s test (*p*<0.05). The HC50 value and their 95% confidence intervals (CI 95%) were obtained by nonlinear regression. For the genotoxicity evaluation it was used the Mann-Whitney U test (*p*<0.05 was considered significant).

## Results

### Identification of volatile compounds in EOM

The essential oil was obtained by hydro distillation from the leaves of *M. sidifolium* with a yield of 0.6% compared to the fresh weight of its botanical material. The total percentage of volatile components identified was of 92.9%, comprising 57 components. The major compounds were fenchone (24.8%), cubebol (6.9%), limonene (5.4%), spathulenol (4.5%), β-caryophyllene (4.6%) and α-cadinol (4.7%) (Table 1).

**Table 1:** Chemical composition of essential oil from aerial parts *of Mesosphaerum sidifolium*

Compounds	% relative	IR
α-pinene	0.1	934
α-fenchene	0.1	946
myrcene	0.4	986
α-phellandrene	1.3	1007
limonene	5.4	1028
γ-terpinene	0.1	1056
terpinolene	0.1	1083
fenchone	24.8	1088
linalol	0.2	1099
*endo*-fenchol	0.3	1118
camphor	0.7	1146
borneol	0.2	1170
4-terpineol	0.4	1178
α-terpineol	0.5	1193
geraniol	0.1	1248
bornyl acetate	1.9	1282
δ-elemene	2.0	1329
α-cubebene	0.1	1372
α -copaene	0.1	1379
β-elemene	0.8	1386
α-gurjunene	0.7	1403
β-caryophyllene	4.6	1416
γ-elemene	0.5	1426
α-guaiene	0.2	1434
*trans*-muurola-3,5-diene	0.3	1442
α-humulene	2.2	1451
duaca-5,8-diene	2.4	1456
*trans*-cadina-1(6),4-diene	0.5	1458
10-*epi*-β-acoradiene	0.2	1462
γ-gurjunene	0.2	1469
γ-muurolene	0.3	1471
germacrene D	0.8	1477
β-selineme	0.2	1484
*trans*-muurola-4(14),5-diene	0.2	1487
biciclogermacrene	1.7	1494
α-muurolene	0.6	1494
γ-cadinene	1.0	1509
cubebol	6.9	1515
δ-cadinene	1.8	1521
α-cadinene	0.2	1532
α-calacorene	0.1	1545
elemol	1.8	1555
palustrol	0.2	1565
spathullenol	4.5	1572
caryophyllene oxide	0.3	1577
globulol	0.4	1589
viridifloral	0.1	1592
guaiol	0.1	1595
ledol	0.3	1599
humulene epoxid II	0.2	1604
1,10-di-*epi*-cubebol	0.2	1610
10-*epi*-γ-eudesmol	0.5	1627
alloaromadendrene epoxid	0.2	1634
*epi*-α-cadinol	1.1	1637
*epi*-α-muurolol	1.4	1640
α-muurolol	0.4	1642
α-cadinol	4.7	1651
shyobunol	0.4	1687

### Cytotoxicity against mice erythrocytes

**Fig. 1:**
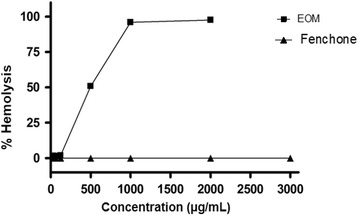
Hemolysis percentage in red blood cells of Swiss mice after treatment with EOM (μg/mL). Each dot represents the average ± SEM of three experiments with three replicates, with 95% confidence interval

EOM displayed a concentration-dependent haemolytic effect. The mean HC50 was 494.9 (494.2–495.6) μg/mL. Differently, fenchone did not induce hemolytic effect up to 3000 μg/mL (Fig. 1).

### Acute preclinical toxicity

EOM 300 mg/kg did not induce death. Following the guideline N. 423 from OECD, we repeated the test using three additional animals, with the same dose. Again, no death was observed. At 300 mg/kg, EOM induced only weak toxicity signs, such as constipation and decreased urination, vocalization and hyperactivity, considered to be without clinical importance, since they disappeared after 60 min of administration. Then, we tested EOM 2000 mg/kg that induced severe symptoms and death of the three animals tested within the first minutes after treatment, therefore this dose was characterized as high toxicity (Table 2). The LD_50_ was estimated approximately in 500 mg/kg.

**Table 2:** Effect of single doses (i.p.) of EOM in mice (*n*=3)

Groups	Dose (mg/kg)	D/T*	Symptoms
Control	-	0/3	None
EOM	300	0/3	Constipation and decreased urination
EOM	300	0/3	Decreased urination, Vocalization and hyperactivity
EOM	2000	3/3	Tremors, convulsions, ataxia, loss of corneal and auricular reflex, cyanosis, loss of muscle tone and no force to grasp

*D/T = Number of dead mice/number of treated mice

### Genotoxicity

EOM only in the highest dose (300 mg/kg) induced an increase in the number of micronucleated erythrocytes in peripheral blood (5.60 ± 0.40), as compared to the control group (2.80 ± 0.37). As expected, cyclophosphamide induced an increase of number of micronucleated erythrocytes (14.50 ± 2.60) (Table 3).

**Table 3:** Number of micronucleated erythrocytes in peripheral blood of mice treated with single doses of EOM and cyclophosphamide (i.p.) (*n*=5)

Groups	Dose (mg/kg)	Number of micronucleated erythrocytes
Control	-	2.8 ± 0.4
Cyclophosphamide	50	14.5 ± 2.6^a^
EOM	150	4.4 ± 0.6
EOM	300	5.6 ± 0.4^a^

Data are presented as mean ± SEM of five animals analyzed by Mann-Whitney U test. ^a^ *p*<0.05 compared to the control group

### In vivo antitumor activity

EOM (100 and 150 mg/kg) induced a significant decrease in tumor weight and volume, and tumor cell total count, when compared to the tumor control group. For fenchone (60 mg/kg), it was observed reduction in all analyzed parameters. Similar results were observed for the standard drug (5-FU, 25 mg/kg). Data are shown in Table 4.

**Table 4:** Effects of EOM, fenchone and 5-FU on tumor volume, weight, and total viable cancer cells in mice (*n*=6) transplanted with Ehrlich ascites carcinoma cells subjected to different treatments (9 days)

Groups	Dose, mg/kg	Tumor volume, mL	Tumor weight, g	Total viable cancer cells, ×10^7^
Tumor Control	-	7.6 ± 0.6	11.9 ± 0.9	339.8 ± 46.4
5-FU	25	0.1 ± 0.0^a^	2.3 ± 1.0^a^	8.5 ± 2.3^a^
EOM	50	4.9 ± 2.2	12.1 ± 1.3	120.1 ± 18.9
EOM	100	0.0 ± 0.0^a^	0.8 ± 0.4^a^	4.4 ± 1.8^a^
EOM	150	0.0 ± 0.0^a^	1.3 ± 0.2^a^	6.9 ± 4.1^a^
Fenchone	30	5.5 ± 1.0	8.4 ± 1.0	228.0 ± 39.9
Fenchone	60	0.8 ± 0.6^a^	3.9 ± 0.9^a^	10.9 ± 4.1^a^

Data presented as mean ± SEM of six animals analyzed by ANOVA followed by Tukey test. ^a^ *p*  <  0.05 compared to tumor control

**Fig. 2:**
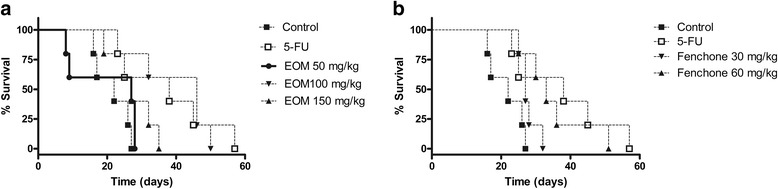
Survival times of female mice inoculated with Ehrlich carcinoma cells and treated with EOM (**a**) and Fenchone (**b**). Data presented as mean ± SEM of six animals analyzed by Kaplan-Meier test

In addition, there was an increase in survival time of all of groups, when compared with the tumor control group, however this effect was more pronounced at 100 mg/kg EOM (Fig. 2a) and 60 mg/kg fenchone (Fig. 2b).

### Analysis of cell cycle

**Fig. 3:**
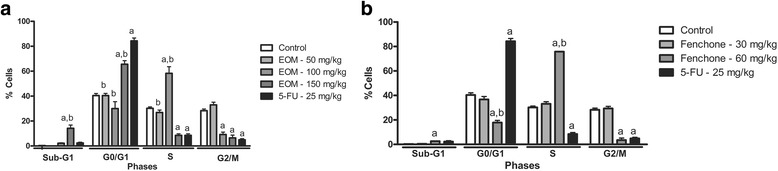
Percentage of Ehrlich ascites carcinoma cells in different phases of the cell cycle after treatment with 5% Tween 80 solution (tumor control), 5-FU (25 mg/kg), EOM (50, 100 and 150 mg/kg) (**a**) and Fenchone (30 and 60 mg/kg) (**b**). Data presented as mean ± SEM of six animals analyzed by ANOVA followed by Tukey test. ^a^ *p*  <  0.05 compared to tumor control. ^b^ *p*<0.05 compared to 5-FU

EOM (100 and 150 mg/kg) exhibited significant changes in the distribution of Ehrlich carcinoma cells at different stages of the cell cycle (Fig. 3a). The results show that EOM 100 mg/kg induced increase in the S phase (58.27%), accompanied by the reduction in G2/M phase (9.1%). At the highest dose (150 mg/kg EOM), it was observed and increase in sub-G1 peak to 14.1% and in G0/G1 phase (65.6%), associated with descrease in S and G2/M phases (13.7% and 6.4%, respectively). For 5-FU, G0/G1 arrest was observed (84.3%), accompanied by the reduction in S (8.4%) and G2/M (4.8%) phases.

Fenchone also induced changes on cell cycle. There was an increase in S phase to 75.8%, associated with decrease in G2/M (3.5%) and G0/G1 (17.8%) phases (Fig. 3b).

### Toxicity evaluation for transplanted mice

EOM (50 mg/kg) produced an increase on water consumption, whereas at 150 mg/kg there was a decrease on feed consumption, when compared to respective healthy and tumor control groups. In addition, EOM (100 and 150 mg/kg) and 5-FU induced significant weight loss in the experimental animals after nine days of treatment. For fenchone (30 and 60 mg/kg), there was only an increase on water consumption (Table 5). In relation to organs index, there was an increase in liver index for tumor control group comparing with health animals; however, EOM reduced this parameter in relation to tumor control group. Fenchone did not restore the normal values for liver index. In addition, fenchone induced an increase in heart and thymus indexes (Table 6).

**Table 5:** Feed and water consumption and weight of animals (*n* = 6) subjected to different treatments (9 days)

Groups	Dose, mg/kg	Water consumption, ml	Feed consumption, g	Gain/loss of weight, %
Healthy animals	-	35.6 ± 1.1	30.9 ± 1.6	12.79 ± 3.0
Tumor control	-	34.6 ± 2.1	29.3 ± 1.5	11.3 ± 1.1
5-FU	25	29.4 ± 1.8	32.8 ± 1.2	−10.1 ± 1.2^a,b^
EOM	50	47.2 ± 2.3^a,b^	33.9 ± 2.9	17.3 ± 4.7^c^
EOM	100	32.5 ± 1.6	30.8 ± 1.6	−8.6 ± 4.3^a,b^
EOM	150	29.4 ± 2.7	23.3 ± 2.1^a^	−12.9 ± 1.9 ^a,b^
Fenchone	30	42.2 ± 1.0^a,b,c^	35.6 ± 0.8	5.6 ± 0.9^c^
Fenchone	60	46.6 ± 1.6^a,b,c^	35.3 ± 0.1	10.23 ± 3.4^c^

Data presented as mean ± SEM of six animals analyzed by ANOVA followed by Tukey test. ^a^ *p* < 0.05 compared to tumor control. ^b^ *p*<0.05 compared to healthy animals. ^c^ *p*<0,05 compared to 25 mg/kg 5-FU group by ANOVA followed by Tukey

**Table 6:** Effects of EOM, fenchone and 5-FU in the mice organ indices (*n* = 6) subjected to different treatments (9 days)

Groups	Dose, mg/kg	Heart index, mg/g	Liver index, mg/g	Kidneys index, mg/g	Thymus index, mg/g	Spleen index, mg/g
Healthy animals	-	4.2 ± 0.3	50.8 ± 2.0	10.9 ± 0.5	3.7 ± 0.5	5.5 ± 0.5
Tumor control	-	3.5 ± 0.2	71.2 ± 3.1^b^	10.6 ± 0.3	2.5 ± 0.2	5.7 ± 0.7
5-FU	25	4.8 ± 0.5	57.4 ± 1.9^a^	12.3 ± 0.3	2.9 ± 0.0	6.3 ± 0.5
EOM	50	3.2 ± 0.5	59.3 ± 2.9 ^a^	9.0 ± 0.7	2.6 ± 0.6	6.5 ± 1.6
EOM	100	4.0 ± 0.1	55.8 ± 1.6^a^	11.5 ± 0.4	3.6 ± 0.4	7.1 ± 0.4
EOM	150	4.2 ± 0.2	56.8 ± 3.0^a^	11.6 ± 0.9	2.4 ± 0.2	7.4 ± 1.2
Fenchone	30	4.0 ± 0,2	71.4 ± 6.3^b^	11.0 ± 0.7	4.1 ± 0.2^a,c^	8.9 ± 1.3
Fenchone	60	4.7 ± 0.4^a^	75.6 ± 1.4^b,c^	13.4 ± 1.0	3.5 ± 0.3	7.9 ± 0.7

Data presented as mean ± SEM of six animals analyzed by ANOVA followed by Tukey test. ^a^ *p* < 0.05 compared to tumor control. ^b^ *p*<0.05 compared to healthy animals. ^c^ *p*<0,05 compared to 25 mg/kg 5-FU group by ANOVA followed by Tukey

EOM induced no significant changes for AST and ALT activity levels. At 150 mg/kg there was only an increase in urea levels, without changes for creatinine levels. On the other hand, fenchone induced significant decrease in liver function parameters, AST and ALT (Table 7).

**Table 7:** Effects of EOM, fenchone and 5-FU on biochemical parameters of peripheral blood of mice (*n*=6) subjected to different treatments (9 days)

Groups	Dose, mg/kg	AST, U/L	ALT, U/L	Urea, mg/dL	Creatinine, mg/dL
Healthy animals	-	283.2 ± 24.9	53.6 ± 6.55	39.0 ± 1.9	0.4 ± 0.0
Tumor control	-	242.0 ± 12.2	67,80 ± 7,11	43.0 ± 7.1	0.3 ± 0.0
5-FU	25	199.3 ± 38.4	39.00 ± 9.51	33.2 ± 1.5	0.51 ± 0.02
EOM	50	217.4 ± 27.4	53.8 ± 11,81	27.5 ± 12.0	0.2 ± 0.0
EOM	100	285.4 ± 22.1	60.3 ± 1.43	45.9 ± 5.7	0.2 ± 0.0
EOM	150	320.2 ± 73.9	44.0 ± 6.86	81.8 ± 8.4^a,b^	0.6 ± 0.2
Fenchone	30	155.8 ± 7.5^a,b^	29,25 ± 2,68^a^	38.8 ± 3.8	0.3 ± 0.0
Fenchone	60	163.8 ± 8.5^a,b^	34.6 ± 5.4^a^	37.8 ± 2.4	0.2 ± 0.0

Data presented as mean ± SEM of six animals analyzed by ANOVA followed by Tukey test. ^a^ *p*<0.05 compared to tumor control. ^b^ *p*<0.05 compared to healthy animals by ANOVA followed by Tukey

Regarding hematological evaluation, changes were observed in tumor control group, comparing with health animals, which included a decrease in red blood cells, hemoglobin and hematocrit. In contrast, EOM increased these parameters in relation to tumor control group, but these values remained as health animals. In addition, there was a leukemoid reaction in tumor control group; nevertheless, EOM (100 or 150 mg/kg) and 5-FU induced a significant reduction in this parameter. Yet, 5-FU induced lymphocyte increase and neutrophil count reduction compared to the tumor control group (Table 8). For fenchone, there was a re-established of the blood count parameters as health animals, comparing to the tumor control group. Similar to EOM, fenchone induced a significant reduction in total leukocytes number (Table 9).

**Table 8:** Effects of EOM and 5-FU on hematological parameters of peripheral blood of mice (*n* = 6) subjected to different treatments (9 days)

Parameters	Healthy animals	Tumor control	5-FU		EOM	
			25 mg/kg	50 mg/kg	100 mg/kg	150 mg/kg
Red blood cells, 10^6^/mm^3^	9.3 ± 0.1	5.8 ± 0.4^b^	9.7 ± 0.2^a^	6.9 ± 0.1	8.1 ± 0.2^a^	9.0 ± 0.3^a^
Hemoglobin, g/dL	14.8 ± 0.2	9.4 ± 0.6^b^	14.9 ± 0.4^a^	10.2 ± 0.7	13.4 ± 0.3^a^	14.7 ± 0.7^a^
Hematocrit, %	43.8 ± 0.5	29.0 ± 1.8^b^	46.6 ± 1.0^a^	40.0 ± 1.6^a^	43.8 ± 1.4^a^	44.2 ± 2.1^a^
MCV, fm^3^	46.6 ± 1.0	49.8 ± 0.6	49.0 ± 0.6	57.6 ± 2.2^a,b^	55.6 ± 1.0^a,b^	48.8 ± 0.6
MCH, pg	15.8 ± 0.5	16.0 ± 0.1	15.4 ± 0.2	14.8 ± 1.1	17.0 ± 0.2	16.2 ± 0.3
MCHC, g/dL	33.7 ± 0.3	34.5 ± 0.8	31.3 ± 0.2	25.7 ± 1.6^a,b^	30.7 ± 0.3	33.2 ± 0.6
Total leukocytes, 10^3^/mm^3^	8.1 ± 0.4	15.1 ± 1.1^b^	3.2 ± 0.1^a^	29.8 ± 8.7^a,b^	3.1 ± 0.4^a^	5.0 ± 1.6^a^
Lymphocytes, %	60.6 ± 4.2	52.4 ± 12.7	90.4 ± 0.9^a^	43.6 ± 9.4	56.6 ± 2.1	51.2 ± 11.9
Neutrophils, %	34.6 ± 4.2	45.4 ± 12.4	7.0 ± 0.7^a^	46.6 ± 8.8	35.6 ± 2.9	47.4 ± 13.9
Monocytes, %	4.4 ± 0.7	2.0 ± 0.5	2.6 ± 0.7	4.0 ± 0.6	2.4 ± 0.5	3.4 ± 0.4
Eosinophils, %	0.4 ± 0.2	0.2 ± 0.2	0.0 ± 0.2	0.2 ± 0.2	0.0 ± 0.0	0.0 ± 0.0

Data are presented as mean ± SEM of six animals analyzed by ANOVA followed by Tukey test. ^a^ *p* < 0.05 compared to tumor control; ^b^ *p* < 0.05 compared to healthy animals by ANOVA followed by Tukey

**Table 9:** Effects of fenchone and 5-FU on hematological parameters of peripheral blood of mice (*n* = 6) subjected to different treatments (9 days)

Parameters	Healthy animals	Tumor control	5-FU	Fenchone	
			25 mg/kg	30 mg/kg	60 mg/kg
Red blood cells, 10^6^/mm^3^	9.3 ± 0.1	5.8 ± 0.4^b^	9.7 ± 0.2^a^	6.9 ± 0.4^b,c^	8.0 ± 0.4^a^
Hemoglobin, g/dL	14.8 ± 0.2	9.4 ± 0.6^b^	14.9 ± 0.4^a^	12.0 ± 0.33^a,b,c^	13.3 ± 0.6
Hematocrit, %	43.8 ± 0.5	29.0 ± 1.8^b^	46.6 ± 1.0^a^	40.2 ± 0.9^a^	45.2 ± 3.3^a^
MCV, fm^3^	46.6 ± 1.0	49.8 ± 0.6	49.0 ± 0.6	59.0 ± 3.5^a,b,c^	59.2 ± 2.2^a,b,c^
MCH, pg	15.8 ± 0.5	16.0 ± 0.1	15.4 ± 0.2	17.2 ± 0.2 ^a,b,c^	16.6 ± 0.3
MCHC, g/dL	33.7 ± 0.3	34.5 ± 0.8	31.3 ± 0.2	28.4 ± 0.9 ^a,b^	31.0 ± 0.7
Total leukocytes, 10^3^/mm^3^	8.1 ± 0.4	15.1 ± 1.1^b^	3.2 ± 0.1^a^	20.2 ± 1.4^a,b,c^	4.0 ± 0.6^a^
Lymphocytes, %	60.6 ± 4.2	52.4 ± 12.7	90.4 ± 0.9^a^	41.6 ± 8.4^c^	64.6 ± 5.6
Neutrophils, %	34.6 ± 4.2	45.4 ± 12.4	7.0 ± 0.7^a^	52.8 ± 8.6^c^	30.0 ± .5.4
Monocytes, %	4.4 ± 0.7	2.0 ± 0.5	2.6 ± 0.7	2.0 ± 0.5	1.4 ± 05^b^
Eosinophils, %	0.4 ± 0.2	0.2 ± 0.2	0.0 ± 0.2	0.0 ± 0.0	0.0 ± 0.0

Data are presented as mean ± SEM of six animals analyzed by ANOVA followed by Tukey test. ^a^ *p* < 0.05 compared to tumor control; ^b^ *p* < 0.05 compared to healthy animals. ^c^ *p*<0,05 compared To the group treated with 25 mg / kg of 5-FU by ANOVA followed by Tukey

**Fig. 4:**
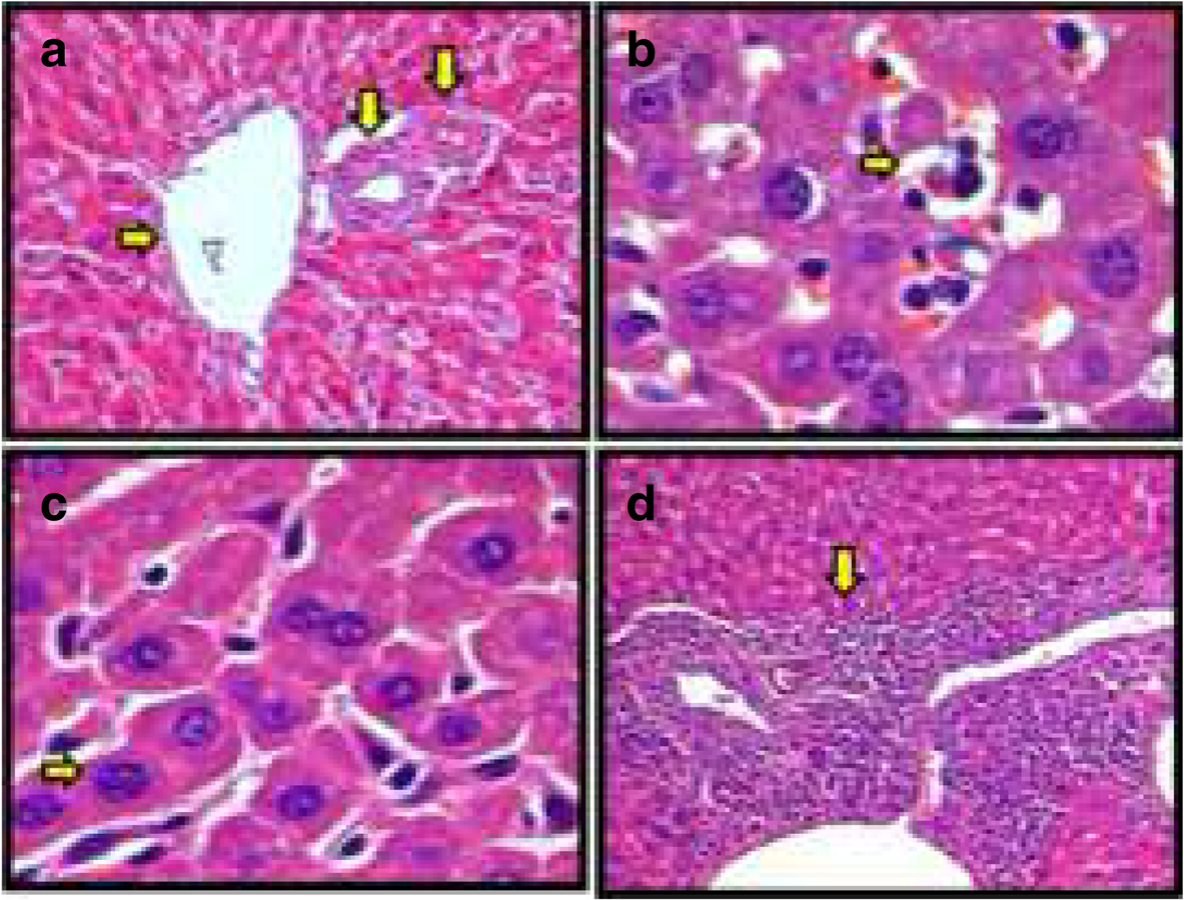
Histopathology of the livers from the different experimental groups: (**a**) *Mesosphaerum sidifolium* essential oil (EOM) (50 mg/kg) - Portal space with a vasculobiliar triad; (**b**) EOM (150 mg/kg) - Anaplastic tumor cells, bordered by necrosis of the hepatic tissue; (**c**) EOM (100 mg/kg) - Reactive hyperplasia of Kuppfer cells; (**d**) 5-FU (25 mg/kg) - Lymphocytes at the moderate level compromising the portal space. Hematoxylin-eosin (100 or 400 x)

No histopathological changes were observed in the kidneys of animals treated with EOM (Data not shown). The histopathological analysis of the livers showed a discrete, reactive lymphocytic, with rare and focal hepatocitolytic activity after treatment with EOM. It was also observed the presence of neutrocyte portite and capsulitis, besides hepatic tissue necrosis (Fig. 4a e b). In addition to these observations, also detected in the tumor control group, the liver of animals treated with EOM presented mild hyperplasia of Kuppfer cells (Fig. 4c). Animals treated with 5-FU experienced hepatic damage greater than those treated with the highest doses of EOM (Fig. 4d).

## Discussion

There are few data in the literature on the chemistry and pharmacology of the genus *Mesosphaerum*, and no reports were founded about *Mesosphaerum sidifolium* yet. The chemical composition described here for EOM is consistent with literature data for volatile constituents from *Hyptis* species [29]. The major component was the bicyclic terpenoid fenchone, which has not reports on antitumor activity. Then, herein we presented the toxicity and antitumor activity of EOM and fenchone.

Anaemia is the most common haematological cancer problem. Considering that chemotherapy can worsen the clinical situation, and this effect can be associated with the hemolysis and/or the inability of the bone marrow to make these cells [30], the cytotoxicity assay with erythrocytes is used to evaluate new drugs toxicity. Natural product due to not presenting hemolytic activity should present CH50 higher than 1250 μg/mL [31]. Then, EOM showed moderate cytotoxic effect in erythrocytes. In contrast, fenchone was considered low toxicity in mice’s erythrocytes. Moreover, studies have documented that essential oils and their chemical constituents may have hemolytic effects [32]. This activity has been primarily related to their ability to interact with the cell membrane due to their lipophilic properties, which alter permeability, promoting hemolysis [33].

The in vivo EOM effects were investigated by acute preclinical toxicology assay, with the aim to establish safe doses to be used in in vivo pharmacological assays. The results showed that EOM has moderate acute toxicity, considering its LD_50_ value 500 mg/kg. In general, if the LD_50_ of the drug is three times more than the minimum effective dose, the substance is considered a good candidate for further studies [34, 35]. Therefore, we used 150 mg/kg of EOM as the highest dose for testing. For fenchone, the dose levels were chosen considering the fenchone percentage in the EOM.

Genotoxicity assays are important for identifying potential carcinogens and mutagens [36]. The micronucleus test has been widely used to test the genotoxicity of several samples, including natural products [37]. Considering that EOM induced increase in micronucleated erythrocytes number only at a dose level two times higher than the antitumor therapeutic dose, it was considered as low toxicity.

In recent decades, several animal models have been developed to investigate the in vivo antitumor activity of new drugs. Here, we used Ehrlich ascites carcinoma cells which is referred to as an undifferentiated carcinoma, and is originally hyperdiploid, has high transplantable capability, no-regression, rapid proliferation, shorter life span, 100% malignancy and does not have tumor specific transplantation antigen [37]. EOM (100 and 150 mg/kg) and fenchone (60 mg/kg) reduced all analyzed parameters (tumor volume and mass, and total viable cancer cells).

The main goal of cancer treatment is to eradicate the disease. However, in situations where cure is impossible, the focus is on improving symptoms and preserving quality of life associated with increased patient survival. New chemotherapeutic agents are associated with increased survival in patients with some types of cancer [38]. In this context, EOM and fenchone induced an important increase in the survival of the animals exposed. Considering that there was no significant difference in the effect of 100 and 150 mg/kg EOM in the parameters tumor volume and weight, and total viable cancer cells, and both doses had similar toxicity profile, we have shown the advantages of 100 mg/kg EOM.

Many anticancer drugs have been isolated from plant species or are based on such substances. In addition, essential oils and their components, especially monoterpenes and phenylpropanoids, have shown antitumor activity [5–7, 39, 40]. Some of the constituents present in EOM are described in the literature as having significant antitumor activity, specifically limonene [41], β-caryophyllene [42].

It is known that cell cycle dysregulation is one of the main characteristics of tumor cells [43]. The induction to the cell cycle arrest has been considered as an important mechanism of the drugs involved in the anticancer treatment [44–46].

For EOM 150 mg/kg and 5-FU treatment, most cells were arrested in the G0/G1 phase, whereas for fenchone, cells arrested in the S phase, which represents a blockage in cell cycle progression. As described in the literature, cell cycle arrest in G0/G1 gives the cells time to repair damage before DNA replication occurs or activating the apoptotic pathway [47]. Furthermore, the essential oil and fenchone at the higher doses induced a significant increase in the sub-diploid population (sub-G1 peak), which is considered as a marker of cell death by apoptosis [48]. The results of the cell cycle analysis corroborate the study of the mechanism of action of many essential oils and their components that stop the cell cycle in the G0/G1 [6] and S phase.

It is well-reported in the literature that the use of antineoplastic drugs can produce numerous adverse effects. Therefore, the use of tests that evaluate possible alterations of the organism homeostasis exposed to chemotherapy is essential. Repeated dose treatment (nine days) differs from the acute toxicity test because it allows evaluating the effects produced by the drug accumulation in the body. According to the results obtained, it is possible to suggest that fenchone exerts weak toxicity considering metabolic parameters, as feed and water consumption, and organ index, since it induced point changes. Regarding EOM, the data show that the essential oil and 5-FU induced weight loss, which is an adverse effect known to many antineoplasic drugs. It was observed an increase in liver index in tumor control group, which was associated with a reactive liver as consequence of tumor implantation in peritoneal cavity. However, EOM, but not fenchone, induced a decrease in liver index, restoring the normality as healthy animals.

Biochemical parameters were evaluated in order to investigate the renal and liver function. The data show EOM induced no toxicity, since the only change observed on urea levels has not clinical significance because the creatinine level was normal. For fenchone, the decrease in liver enzymes (AST and ALT) levels suggest hepatic damage, since severe and persistent changes such as fibrosis may produce little hepatocellular extravasation, resulting in normal or decreased levels of these enzymes [49, 50]. However, additional studies of liver function and histology should be performed to clarify that effect.

Concerning the hematological parameters, it was observed that the tumor implantation alone was able to induce anemia, which is a cancer characteristic. EOM and fenchone reversed this effect, reestablishing the normal levels of blood counts, showing that these natural products even in the presence of the tumor do not interfere with these parameters and therefore does not induce toxicity at the blood cell level as well as hematopoiesis. Inoculation of the tumor alone causes a leukemoid reaction in the transplanted animals, which can be observed by the increase of total leukocytes in the transplanted control compared to the healthy one. These data corroborate with literature data that describe this type of reaction in the presence of the tumor [51–54]. Nevertheless, EOM and fechone, as 5-FU, induced immunosuppression associated with reduction on total leukocytes number, but this effect is not limiting for its study because most of the antineoplasic drugs induce this effect.

In this study, the histopathological findings for the kidneys support the idea that EOM has low renal toxicity, agreeing to biochemical data. In the liver, the changes observed in the animals treated with EOM were characteristic of systemic neoplastic disease with repercussion in the liver, considering the implantation of tumor cells in peritoneal cavity. However, some studies have shown that these changes occurring in all treated groups are related to poor hepatotoxicity, and drug withdrawal or dose adjustment usually leads to rapid improvement and damage reversal [55, 56].

## Conclusions

Considering all data showed EOM caused in vivo cell growth inhibition on Ehrlich ascites carcinoma model by inducing cell cycle arrest, and this activity was associated with the presence of fenchone, its major component. Regarding to toxicological parameters, EOM induced weak toxicity considering biochemical and histological parameters, and induced immunosuppression. For fenchone, the data showed hepatotoxicity and immunosuppression, which is the most common undesirable effect of antineoplasic drugs.

## Abbreviations

5-FU: 5-Fluorouracil
ALT: Alanine aminotransferase
ANS: Autonomic Nervous System
AST: Aspartate aminotransferase
CEUA: Animal Ethics Committee
CI: Confidence Interval
CNS: Central Nervous System
DMSO: Dimethylsulfoxide
EOM: *Mesosphaerum sidifolium* essential oil
GC: Gas Cromatography
GC-MS: Gas Chromatography - Mass Spectrometry
Hb: Hemoglobin level
HC_50_: Concentration producing 50% hemolysis
Hct: Hematocrit
i.p.: Intraperitoneal route
LD_50_: Dose responsible for the death of 50% of the experimental animals
MCH: Mean corpuscular hemoglobin
MCHC: Mean corpuscular hemoglobin concentration
MCV: Mean corpuscular volume
PBS: Phosphate-buffered saline
RBC: Red blood cell count
UFPB: Federal University of Paraíba
