# Erratum to: toxicity and antitumor potential of *Mesosphaerum sidifolium* (Lamiaceae) oil and fenchone, its major component

**DOI:** 10.1186/s12906-017-1875-0

**Published:** 2017-07-14

**Authors:** Thaísa Leite Rolim, Déborah Ribeiro Pessoa Meireles, Tatianne Mota Batista, Tatyanna Kelvia Gomes de Sousa, Vivianne Mendes Mangueira, Renata Albuquerque de Abrantes, João Carlos Lima Rodrigues Pita, Aline Lira Xavier, Vicente Carlos Oliveira Costa, Leônia Maria Batista, Marcelo Sobral de Abrantes, Marianna Vieira Sobral

**Affiliations:** 1 Programa de Pós-graduação em Produtos Naturais e Sintéticos Bioativos, Centro de Ciências da Saúde, Universidade Federal da Paraíba, João Pessoa, Paraíba 58051-970 Brazil; 2 Departamento de Ciências Farmacêuticas, Universidade Federal da Paraíba, João Pessoa, Paraíba 58051-970 Brazil

## Erratum

After the publication of this article [1] it came to our attention that the author list was incorrect. The author Josean Fechine Tavares was included due to an error during the production process. The correct author list is shown in this erratum updated in the original article.
